# Investigating age-related differences in semantic control mechanisms involved in creative cognition

**DOI:** 10.3758/s13421-025-01753-6

**Published:** 2025-07-11

**Authors:** Tanvi Patel, Sarah E. MacPherson, Paul Hoffman

**Affiliations:** Human Cognitive Neuroscience, Department of Psychology, School of Philosophy, Psychology & Language Sciences, University of Edinburgh, 7 George Square, Edinburgh, EH8 9JZ UK

**Keywords:** Aging, Creative thinking, Semantic memory, Executive function

## Abstract

Creative thinking is a complex, higher-order ability that draws on multiple cognitive systems. However, the contribution of specific semantic control processes to creativity remains unclear. The current study had two goals: First, we investigated how individual differences in semantic knowledge and control contribute to divergent and convergent styles of creative thinking, beyond the involvement of domain-general executive functions. Second, we explored whether there were age-related differences in semantic and executive abilities, and if these differences influenced the ability to think creatively. Specifically, we examined the role of the two components of semantic control: controlled retrieval and semantic selection. In our study, 63 younger adults and 64 older adults completed semantic, executive, and creative thinking measures. Younger adults demonstrated better executive functioning, while older adults exhibited superior semantic knowledge, controlled retrieval, and convergent thinking abilities. Crucially, there were no age differences across several divergent thinking metrics: automated originality scoring, human ratings, or uniqueness. Regression analyses indicated that semantic knowledge and updating executive ability influenced convergent thinking abilities across both age groups. In contrast, semantic control abilities were predictive of divergent thinking skills, but only in the younger group. Our results emphasize the key role of the semantic system in creative thought, and, critically, indicate that divergent and convergent thinking may rely on different aspects of semantic cognition. Moreover, the recruitment of these abilities varies across the lifespan, in line with increased knowledge reserves and declines in executive control seen in older adults.

**Supplementary Information:**

The online version contains supplementary material available at 10.3758/s13421-025-01753-6.

## Introduction

Creative thinking, the process of generating original and useful ideas, draws from a diverse toolbox of cognitive resources. One such resource is the semantic memory system that stores our vast repository of knowledge about the world, providing the foundation for new ideas (Abraham & Bubic, 2015; Benedek et al., 2017; Gerver et al., 2023). However, effective use of this knowledge requires semantic control mechanisms—cognitive processes that regulate how concepts are retrieved, manipulated, and selected to align with task-related goals (Badre et al., 2005). While the literature has established a strong link between creativity and various facets of domain-general executive control (e.g., Beaty & Silvia, 2012; Beaty et al., 2014a, 2014b; Benedek & Neubauer, 2013; Lee & Therriault, 2013; Nusbaum & Silvia, 2011), the contribution of specific *semantic* control processes remains unexplored. The present study aims to bridge this gap by examining how semantic control processes contribute to creativity, over and above domain-general executive functions.

This question is particularly relevant in the context of aging, given the well-documented changes in cognitive systems across the lifespan. While older adults face declines in general executive functioning, their semantic knowledge and certain aspects of semantic control are well-preserved (Hoffman, 2018; Kavé & Halamish, 2015; Kavé & Yafé, 2014; Park et al., 2002; Verhaeghen, 2003; Wu et al., 2022). Notably, despite declines in executive control, older adults retain the ability to think creatively (Fusi et al., 2021), which may suggest a compensatory role of semantic abilities in supporting creativity in later life. To examine this, the present study investigated the contributions of semantic knowledge and control abilities to creative thinking in both younger and older adults, exploring whether these groups engage distinct cognitive mechanisms in pursuit of creative goals.

### The cognitive basis of creativity

Creativity is often described as involving both *divergent* thinking (the ability to generate multiple possible responses to an open-ended constraint) and *convergent* thinking (the process of homing in on a single solution to a goal-directed problem; Guilford, 1967). The literature has often treated divergent and convergent thinking as opposing forces, with the divergent thinking capabilities taken as being more representative of creative potential (Eysenck, 2003). However, it is likely that both these modes of thinking are engaged during the creative process, with divergent thinking broadening the search space and convergent thinking narrowing it to select the best solution (Cortes et al., 2019). The interwoven nature of divergent and convergent thinking is echoed in dual-process models that suggest that the creative process involves distinct stages of idea generation and evaluation (Barr et al., 2015; Benedek & Jauk, 2018; Finke et al., 1992; Kleinmintz et al., 2019; Sowden et al., 2015; Volle, 2018). As we review below, these phases of generation and evaluation rely on different cognitive mechanisms: Idea generation requires the bottom-up activation of concepts from memory, while the evaluation and selection of ideas requires top-down executive processing.

### Content and organisation of semantic knowledge

When generating new ideas, individuals draw from various sources, including their conceptual knowledge about the world (semantic memory) and memories of autobiographical events (episodic memory; Benedek et al., 2023). While there is evidence linking creativity with episodic memory function (Dewhurst et al., 2011; Thakral et al., 2021), a recent meta-analysis suggested that semantic memory is more strongly related to both divergent and convergent thinking, indicating conceptual knowledge serves as crucial raw material for creative thought (Gerver et al., 2023).

Associative theories of creativity (Mednick, 1962) emphasise that beyond content, the organisation and structure of semantic memory play a key role in shaping creative thought. According to Mednick (1962), creativity refers to the construction of new associative links between concepts, with more distant combinations resulting in more creative outcomes. Under this framework, concepts are organized in associative hierarchies (i.e., the arrangement of concepts based on the strength and type of associations), and individual differences in creative ability arise from the structure of these associative hierarchies. As in classic spreading activation models (Collins & Loftus, 1975), when a concept is activated, there is an automatic spread of activation to related concepts through these associative links. Creative individuals have ‘flatter’ associative hierarchies, where concepts are linked to a wide range of common and remote associations with similar associative strengths, enabling them to easily access and combine more distant associations. In contrast, less creative individuals have ‘steeper’ hierarchies characterized by fewer, stereotypical associations between concepts, making it difficult to generate new ideas.

Modern computational network-based approaches have been instrumental in testing Mednick’s theory: numerous studies have examined how the structure of the semantic system is related to divergent thinking at the group (Kenett et al., 2014, 2018) and individual level (Benedek et al., 2017; He et al., 2021). These studies suggest that creative individuals have densely connected and flexible semantic networks with shorter path lengths and lower modularity, allowing for more efficient retrieval and combination of distant concepts (Kenett, 2018; Kenett & Austerweil, 2016; Kenett & Faust, 2019; Kenett et al., 2014, 2018). Recent work has indicated that these network structures are also seen in individuals with high convergent thinking ability (Luchini et al., 2023), indicating that flexible semantic networks may facilitate both divergent and convergent thinking.

While such networks provide a broad range of associations to fuel idea generation, they may also lead to interference and potentially affect *quality* of ideas (Beaty et al., 2023). Moreover, in a direct test of Mednick’s hypothesis, Benedek and Neubauer (2013) found no differences in the organisation of associative hierarchies between groups of low and high creative individuals (e.g., both groups are likely to respond ‘*car*’ when cued with ‘*street*’). Notably, while the highly creative individuals did produce more uncommon responses, this was primarily driven by their ability to produce more responses overall, allowing them to reach more distant concepts faster. Together, these findings imply that creativity may not stem solely from differences in associative structure but also from control abilities that enable efficient retrieval and manipulation of semantic memory.

### Semantic control: Regulating knowledge use

New ideas do not simply occur—they are formulated through the manipulation and recombination of our existing semantic knowledge. However, a given concept may spark a range of different associations, only a subset of which is relevant for the task at hand. Thus, to be creative, participants must avoid stereotypical or irrelevant associations, engage in a goal-directed search for ideas, and evaluate these ideas in line with task criteria. It follows that, in addition to having larger, more densely connected semantic networks, creative individuals may be more skilled at regulating how they access and use this knowledge. These goal-directed retrieval and selection processes are often referred to as ‘semantic control’ abilities.

Semantic control mechanisms refer to those processes that regulate the retrieval, manipulation and use of semantic knowledge in a context and task-appropriate way (Badre & Wagner, 2007; Badre et al., 2005; Hoffman et al., 2018; Lambon Ralph et al., 2017). Two specific kinds of semantic control process have been proposed: *controlled retrieval* and *semantic selection* (Badre & Wagner, 2007; Badre et al., 2005). Controlled retrieval processes refer to the goal-directed search through semantic memory when an automatic spread of activation does not arrive at the desired outcome, and semantic selection processes resolve competition between multiple competing representations based on current task requirements. Each of these elements of semantic control may differentially contribute to creative thought. During the idea-generation phase, controlled retrieval processes may support the formation of novel conceptual combinations by facilitating access to less common semantic associations. Meanwhile, semantic selection abilities may be important for inhibiting inappropriate associations when evaluating candidate responses. The neuroimaging literature supports the role of the semantic control system in creative thought: Brain regions associated with semantic control have been associated with divergent thinking (Cogdell‐Brooke et al., 2020) and the generation of unusual links between words (Krieger-Redwood et al., 2023). However, the role of controlled retrieval and semantic selection mechanisms in divergent and convergent thinking has not been tested at a behavioural level.

Semantic control functions are related to, but distinct from, domain-general executive processes that allow individuals to plan and executive goal-directed behaviour (Diamond, 2013). At the neural level, semantic processing has been found to engage domain-general executive control networks as well as specialized semantic control regions, particularly those responsible for retrieval (Bourguignon & Gracco, 2019; Jackson, 2021). Similarly, behavioural evidence indicates that while semantic selection is correlated with general executive abilities, controlled retrieval processes are not (Hoffman, 2018). Thus, semantic control processes may make a unique contribution to creative thought, beyond domain-general executive control.

### Creativity and domain-general cognitive control

Individual differences research has put forth a strong case for the role of executive control in creativity, bolstering controlled-attention theories of creativity (Beaty, Beaty & Silvia, 2012, 2013; Beaty, Silvia, et al., 2014a, 2014b; Benedek et al., 2012, 2014; Cheng et al., 2016; Nusbaum & Silvia, 2011; Palmiero et al., 2022; Zabelina et al., 2019). In line with this, both divergent and convergent thinking have been consistently linked to facets of general intelligence, specifically crystallized (accumulated knowledge) and fluid intelligence (problem-solving capabilities independent of prior knowledge; Cho et al., 2010; Ellis et al., 2021; Gerwig et al., 2021; Lee & Therriault, 2013; Nusbaum & Silvia, 2011; Silvia & Beaty, 2012).

Considerable work has examined how divergent thinking is supported by the core executive functions (Miyake et al., 2000): *inhibition* (suppression of dominant but irrelevant information), *updating* (monitoring and manipulating of information in working memory), and *shifting* (flexibly switching between relevant task sets). However, a review of 57 studies suggests that the pattern of associations is inconsistent, varying with methodological choices such as task type, scoring method and how specific executive functions are measured (Palmiero et al., 2022). Updating has shown the most consistent positive associations with divergent thinking, especially in terms of idea fluency (Batey & Furnham, 2006; De Dreu et al., 2012; Nusbaum & Silvia, 2011). Inhibition showed a more variable relationship to divergent thinking, with studies highlighting both positive (Benedek et al., 2012; Zabelina et al., 2012) and negative (Cheng et al., 2016; Radel et al., 2015) associations. This implies that divergent thinking may require flexible inhibitory control, depending on whether tasks require the filtering out of irrelevant information, or broad associative thinking (Vartanian, 2009). Finally, though shifting has been proposed as being vital to accessing different semantic categories in the search for original ideas, few studies have examined this link, with inconclusive results (Benedek et al., 2014; Zabelina et al., 2019).

Interestingly, working memory also plays a role in convergent thinking. Numerous studies have demonstrated a strong relationship between convergent thinking and working memory capacity (Chein & Weisberg, 2014; Ellis & Brewer, 2018; Ellis et al., 2021; Gerver et al., 2023; Lee & Therriault, 2013). Together, this suggests that the ability to maintain and manipulate information may be integral to both divergent and convergent thinking, allowing individuals to hold multiple pieces of information in mind while searching for and evaluating potential ideas.

### Creativity in aging

So far, we have argued that a diverse set of cognitive systems contribute to creative thought: stored representations of semantic knowledge, specific control processes that act on these semantic representations, and domain-general executive and cognitive functions. Each of these systems are associated with age-related changes, which may impact creativity in later life. Older people possess larger stores of knowledge and a rich vocabulary, accrued from their education and life experience (Kavé & Halamish, 2015; Kavé & Yafé, 2014; Park et al., 2002; Salthouse, 2004; Verhaeghen, 2003; Wu et al., 2022). Despite this, older adults have more segregated, less efficient, and less flexible semantic networks (Cosgrove et al., 2021). These network differences could arise from differences in semantic search abilities across age groups. Indeed, specific aspects of semantic control are differentially affected by age; older adults demonstrate preserved controlled retrieval abilities (at least for verbal knowledge) but are less accurate when the semantic selection demands are high (Hoffman, 2018; Hoffman & MacPherson, 2022; Wu & Hoffman, 2022; Wu et al., 2022). Additional age-related declines are seen across core executive abilities where older adults perform worse on tests of executive functioning, exhibiting large magnitudes of differences for all subdomains, except for updating (Maldonado et al., 2020). Finally, there are age-related differences in facets of intelligence, with increased crystallized knowledge and decreased fluid reasoning abilities (Ryan et al., 2000).

Crucially, there is little understanding of how age-related changes in these cognitive systems affect creativity in later life, as most of the research on creativity has focused on young adult populations and the small body of work looking at creative thinking in aging has found inconsistent results. Some studies have indicated that the development of creative thinking across the lifespan assumes an inverted U-shape where divergent thinking abilities peak before middle age (40–55 years) and then decline (the ‘peak-decline’ hypothesis; Massimiliano, 2015; Palmiero et al., 2017; Simonton, 1999). However, more recent evidence suggests that creative thinking is preserved in older adults (Madore et al., 2016), especially if mediating factors like time (Foos & Boone, 2008) and working memory (Leon et al., 2014) are considered. Indeed, a systematic review of 16 papers from 1970 to 2018 suggests that there is a complex relationship between aging and divergent thinking (Fusi et al., 2021): Aging has differential effects on verbal and visual creativity, with only verbal divergent thinking abilities preserved in older adults (Colautti et al., 2023). In a similar vein, the limited work examining convergent thinking in aging suggests that there are no age-related declines in performance (Alcock et al., 2024), although the underlying cognitive mechanisms supporting this preservation remain unclear.

Thus, in the present study, we collected a wide range of semantic, executive, and creative thinking measures from younger and older adults, focusing on two key questions. First, we examined how individual differences in semantic knowledge and control predict creative abilities, and whether these semantic abilities contribute to creativity beyond domain-general executive functioning. Second, we investigated variation in semantic and executive abilities between younger and older adults, and whether these age-related differences can account for performance on creative tasks. To our knowledge, this study is the first to examine how the specific semantic control components—controlled retrieval and semantic selection—differentially relate to convergent and divergent thinking, and whether age-related changes in these abilities affect creative output.

## Methods

### Transparency and openness

In the following section, we have outlined information needed to reproduce the analyses: sample-size estimation, data exclusions, measures used, and scoring methods. The design and analyses for this study were not preregistered. All analyses were done in R (Version 4.3.2; R Core Team, 2023), using the following packages: *tidyverse* (Version 2.0; Wickham et al., 2019), *lme4* (Version 1.1.35.1; Bates et al., 2015), *ggeffects* (Version 1.5.1; Lüdecke, 2018), *patchwork* (Version 1.1.3; Pedersen, 2023), and *wesanderson* (Version 0.3.7; Ram & Wickham, 2023). Data and code for the study can be accessed online (https://osf.io/ev7qm/?view_only=4109221c93264aa2ba63c885cf1b1ee3) and study materials can be provided upon request. The study received ethical approval from the Psychology Research Ethics Committee (Ref No: 164–1920/6). In accordance with the ethical requirements, participants were informed of their rights, and informed consent was acquired.

### Participants

One hundred and twenty-seven older (*n* = 64) and younger adults (*n* = 63) participated in the study. The older group consisted of adults between the ages of 60 and 90 years, recruited through the Volunteer Panel at the Psychology Department, and compensated via a prize draw with gift vouchers. The younger group included students ages 18–35 years from undergraduate psychology courses, who were compensated with course credit. All participants were native English speakers, defined as having learnt English before the age of 5 years. Participants were asked to report if they had ever experienced neurological trauma or illness, or a psychological disorder. Four participants in the older adult group were excluded due to a history of neurological illness, and three participants in the younger adult group were excluded due to colour blindness (*n* = 2) and a diagnosis of attention-deficit/hyperactivity disorder (*n* = 1).

The final sample consisted of 120 people, with 60 people each in the older (41 women; *M*_Age_ = 68.2 years, *SD*_Age_ = 5.20) and younger (49 women; *M*_Age_ = 18.7, *SD*_Age_ = 1.49) groups. A sensitivity analysis indicated that the minimum correlation between two variables that we could reliably detect (with 80% power) was *r* = 0.25 for analyses conducted in each group separately (*N* = 60) and *r* = 0.18 for analyses of both groups combined (*N* = 120). This is based on a power analysis conducted in G*Power 3 (Faul et al., 2007), determining that a sample of 60 people in each group would be sufficient. Data were collected between September 2021 and January 2022 at a university in the UK.

### Procedure

The experiment comprised three parts. In Part 1, participants were tested online, via a 1–1 video call with the experimenter on Zoom. This segment took approximately 1 h and consisted of six tasks, presented in the following order: (1) Digit Symbol Matching Task; (2) Series Completion Task; (3) *N*-back task; (4) Colour-Word Interference Task; (5) Alternate Uses Task; and (6) Number Letter Task.

Following the completion of Part 1, participants were asked to complete Parts 2 and 3 online on their own. Both parts were hosted on the Testable website and lasted approximately 20–25 min. Part 2 consisted of the semantic tasks: (1) Spot the Word; (2) Synonyms; and (3) Semantic Control; while Part 3 included: (1) Remotes Associates Test; (2) Kaufman Domains of Creativity (K-Docs); and (3) Inventory of Creative Activities and Achievements (ICAA).

### Measures of intelligence

We included measures indexing facets of intelligence as outlined in the Cattell-Horn-Carroll (CHC; Cattell, 1971; Schneider & McGrew, 2018) model, specifically speed of processing (i.e., the speed of cognitive operations; ‘Gs’) and fluid intelligence (i.e., reasoning abilities; ‘Gf’). Both speed of processing (Forthmann et al., 2019a, 2019b) and fluid intelligence (Gerwig et al., 2021; Nusbaum & Silvia, 2011; Nusbaum et al., 2014) have been found to be significant predictors of divergent thinking in the literature, warranting their inclusion as covariates in the current analysis. Importantly, speed of processing is a cognitive ability that is preferentially affected by aging (e.g., Salthouse, 1996), making it particularly relevant to the present study.

#### Speed of reasoning: Digit Symbol Matching Task (DSMT)

To measure speed of processing, the Digit Symbol Matching Task from the Wechsler Adult Intelligence Scale-III (Wechsler, 1997) was adapted to a computerized format. Participants were presented with a key consisting of the numbers from 1 to 9, each matched with a corresponding symbol. This key remained on screen for the duration of the experiment, minimizing any memory demands. Participants were then presented with one single symbol at a time, in the centre of their screen, and had to match the symbol to its corresponding number by pressing the correct number key on their keyboard. There were 90 possible stimulus items, and the final score on the task was calculated as the total number of correct responses (i.e., correctly matched symbol/number combinations) generated in a 90-s period. Higher scores indicated faster processing speed. The DSMT has good construct validity, correlating strongly with other tasks that involve perceptual or processing speed (Salthouse, 2000; Sliwinski & Buschke, 1999). Additionally, the DSMT has high test–retest reliability (*r* = 0.82–0.87) across different age groups, supporting its stability over time (Wechsler, 1997). Importantly, previous work using the DSMT has reported substantial correlations with age (ranging from *r* = − 0.46 to − 0.77; e.g., Hoyer et al., 2004; Salthouse, 1992), supporting its use as a tool to assess age-related changes in cognitive processing speed.

#### Fluid intelligence: Series Completion Task

To measure fluid nonverbal reasoning, we used a test similar to Raven’s Progressive Matrices (Raven & Raven, 2003): the Series Completion Task (Scale 3, Form B) from the Cattell Culture Fair tests (Cattell, 1943). Participants were presented with a series of line drawings that changed according to a certain rule and had to choose the final image in the sequence. The final fluid reasoning score reflected the total number of correct responses made within 3 min (from a maximum of 13). The Series Completion Task is a well-validated measure of fluid intelligence, demonstrating strong internal consistency across all items (α = 0.91), and high stability over time with test–retest reliability values ranging from 0.82 to 0.85 (Krug, 1973). Regarding construct validity, factor analytic studies confirm that the Series Completion Task loads strongly onto the general intelligence (g) factor, aligning it with reasoning and problem-solving abilities (Cattell & Gibbons, 1968, as cited in Krug, 1973). The task also shows moderate to strong correlations (*r* = 0.51 to 0.64) with Raven’s Progressive Matrices, supporting its convergent validity as a measure of fluid intelligence (MacArthur & Elley, 1963, as cited in Krug, 1973; Wrightstone, 1958). Given these psychometric properties, the task is highly suitable for assessing nonverbal problem-solving skills independent of crystallized knowledge.

### Measures of executive functioning

Three tests were used to measure the core executive functions of updating, inhibition, and shifting.

#### Updating: N-back Task

Updating was indexed by the *N*-back task, based on the version used by Vaughan et al. (2008). The 2-back version was selected, as a meta-analysis by Bopp and Verhaeghen (2018) found that 2-or-more back tests are more sensitive to age effects than the 1-back. Participants were shown a sequence of letters (in white) in the centre of a black screen. They were told to pay attention to and remember the first two letters of the sequence, presented at a rate of 2,000 ms. From the third letter, participants had to respond by indicating whether the letter they saw currently was the same letter as the one presented two places back in the sequence. Participants responded by a button press, and presentation was self-paced (the next trial appeared only after the participant responded to the previous trial). There were five blocks of 20 trials (100 trials in total), presented in the same order to all participants, with a 50:50 ratio of matching to nonmatching trials. The average proportion of correct responses across the five blocks were used as an index of performance. The visuoverbal *N*-back demonstrates strong test–retest reliability for RT measures of 3-back items (*r* = 0.85) and adequate reliability for accuracy measures (*r* = 0.69; Soveri et al., 2018). Unlike static storage tasks, the *N*-back primarily measures dynamic working memory updating, as it correlates weakly with change detection tasks but strongly predicts transsaccadic memory performance (Frost et al., 2021). Its ability to index online monitoring and maintenance processes makes it a good measure of working memory updating.

#### Inhibition: Colour-Word Interference Task

To measure inhibition, we used the Inhibition and Inhibition/Switching subtests of the Colour-Word Interference Task (CWIT) from the Delis-Kaplan Executive Function System (D-KEFS; Delis et al., 2001), as they show greater age-related declines than the colour naming (CWIT-1) and word reading (CWIT-2) trials, making them more sensitive to cognitive ageing effects (Adólfsdóttir et al., 2017). For the *Inhibition* subtest, participants were shown a series of colour words written in different coloured ink. Participants were told to inhibit their dominant response to read out the colour word and to name the ink colour instead. They were presented with 50 words and the total score was the total time taken to read all stimulus words. The *Inhibition/Switching* subtest was similar but had an additional rule: If there was a box around the word, the participants had to read out the actual word instead of the ink colour. The time taken for participants to respond to 50 stimulus items was recorded. The final inhibition score was derived by averaging performance across the *Inhibition* and *Inhibition/Switching* subtests.

Higher scores indicated poorer performance (i.e., reduced inhibitory control). The CWIT exhibits good psychometric properties: the Inhibition and Inhibition/Switching subtests demonstrated moderate to high internal consistency using split-half reliability (*r* = 0.72–0.90) and moderate to high test–retest reliability (*r* = 0.72 and *r* = 0.68, respectively; Delis et al., 2001; Homack et al., 2005).

#### Shifting: Number–Letter Task

The Number–Letter Task (Miyake et al., 2000; adapted from Rogers & Monsell, 1995) was used to index shifting. A square with four quadrants was presented in the middle of the screen, and a number–letter pair (e.g., ‘G2’) was presented in one of these four quadrants. The task consisted of three blocks: (1) *Block 1*: The Number–Letter combination appeared only in the top quadrants and participants had to indicate whether the number was odd (3, 5, 7, 9) or even (2, 4, 6, 8); *Block 2:* The Number–Letter combination only appeared in the bottom quadrants and participants had to indicate whether the letter was a vowel (A, E, I, U) or a consonant (G, K, M, R); (3) *Block 3*: The Number–Letter combination first appeared in the top left quadrant and then rotated through each quadrant in a clockwise direction. If the Number–Letter combination was in the top quadrants, participants had to respond to the number (i.e., say whether the number was odd or even) but if it was in the bottom quadrants, participants had to respond to the letter (i.e., say whether the letter was a vowel or a consonant). Participants were given 10 practice trials for each block. Blocks 1 and 2 consisted of 32 trials each and did not require task switching; Block 3 consisted of 64 trials, and the trials in the top left and bottom right quadrants required switching between number and letter tasks (32 trials). The shifting cost was calculated as the difference between the average RTs for switch trials in Block 3 and the average RT for no-switch trials in Blocks 1 and 2. The Number–Letter task has been widely used as a measure of task-switching ability and demonstrates strong psychometric properties. Test–retest reliability is moderate to high for switch trials (*r* = 0.73) and for switching costs (*r* = 0.68; Soveri et al., 2018). Internal consistency is also high, with Cronbach’s α = 0.91 for switch trials and α = 0.92 for nonswitch trials (Friedman et al., 2016). In terms of validity, the task correlates well with other measures of shifting, including the Colour-Shape Task (*r* = 0.41) and the Category-Switch Task (*r* = 0.48), supporting its construct validity as an index of cognitive flexibility and mental set shifting (Friedman et al., 2016).

### Measures of semantic knowledge

#### Spot the Word Task

To evaluate the breadth of participants’ store of semantic information, they completed the Spot the Word (STW) test from the Speed and Capacity of Language Processing battery (Baddeley et al., 1992). In the STW test (60 trials), participants were shown a pair of response options (e.g., *toaster–flumpter*) consisting of a word, and a phonologically and orthographically plausible nonword, and had to pick the real word. The STW has good internal reliability (α = 0.83), and correlates strongly with performance on the Mill Hill vocabulary scale (*r* = 0.86**)** as well as the National Adult Reading Test (NART; *r* = 0.60), indicating that it is an effective index of verbal knowledge (Baddeley et al., 1993).

#### Synonyms Task

To test the breadth of semantic knowledge, we used a version of the Synonyms Task adapted from the Mill Hill vocabulary test (Hoffman, 2018; Raven et al., 1989). On the Synonyms Task (44 trials), the participants were presented with a probe word (e.g., *run*) and four response options (*turn, dash, sigh, expire*) and had to match the probe with its synonym (*dash*). Following previous studies (Hoffman, 2018; Hoffman & MacPherson, 2022), the percentage correct responses on the SWT and Synonyms Task were combined to form a composite score indexing semantic knowledge.

### Measures of semantic control

Participants were tested on the two forms of semantic control, using tasks that have been previously employed in the literature (e.g., Badre et al., 2005; Hoffman, 2018; Whitney et al., 2011; Wu & Hoffman, 2022).

#### Controlled retrieval

Global semantic association trials were used to probe participants’ ability to engage in controlled retrieval of less salient semantic information. Participants had to choose the semantic associate of a cue word (e.g., *pebble*) from a set of four alternatives (e.g., *letter, cat, stone, person*). This association was manipulated to be either strong (e.g., *town–city*) or weak (e.g., *iron–ring*), with the weak associations requiring more controlled retrieval of appropriate semantic information to identify the relevant semantic link. There were 24 strong and 24 weak trials over the four blocks of global trials. The proportion of correct responses on the weak trials was taken as a measure of controlled retrieval ability.

#### Semantic selection

Feature-based association trials were used to examine the extent to which participants could resolve competition between multiple semantic alternatives (Thompson-Schill et al., 1997). Participants were presented with a probe (e.g., *pepper*) and four response options (e.g., *ice, tar, salt, cabbage*). They had to match the probe word with a target based on a particular item property (here, colour). On congruent trials, the probe and target were matched on the relevant feature and had a strong semantic association, placing low selection demands on participants as the automatic activation of semantic associations would prompt the correct response (e.g., *strawberry–raspberry*). On the incongruent trials, the probe and target did not have any pre-existing association (e.g., *pepper–tar*), requiring participants to focus specifically on feature-relevant attributes shared by the cue and each of the possible responses. These incongruent trials included foils which were strongly related to the target word (*pepper–salt*), placing further demands on semantic selection processes by necessitating the inhibition of dominant but irrelevant knowledge. Participants were informed which feature they should be focusing on at the start of each block (size or colour). There were 24 congruent and 24 incongruent trials over the 4 blocks of feature-based trials and the proportion of correct responses on the incongruent trials was taken as a measure of semantic selection ability.

### Measures of creativity: Self-report

While our primary interest in the study was the relationship between cognitive abilities to performance-based measures of creative thinking, we included two self-report measures of creativity to assess the degree to which the effects transferred to creative behaviours in everyday life.

#### Creative self-beliefs: Kaufman Domains of Creativity (K-Docs)

The K-Docs (Kaufman, 2012) is a self-report measure that probes beliefs about one’s own creative abilities. The K-Docs presents participants with a list of 50 creative behaviours, covering different domains such as Self/Everyday, Scholarly, Performance, Mechanical/Scientific, and Artistic creativity. Participants are asked to rate their creative ability for each act in comparison to their peers, on a 5-point Likert scale from 1 (*much less creative*) to 5 (*much more creative*)*.* The possible scores for this measure range between 50 and 250, where the higher the score, the higher the creative abilities. The K-Docs exhibits strong internal consistency, with a pooled Cronbach’s α of 0.904 (Sen & Yörük, 2023), and test–retest reliability coefficients ranging from 0.76 to 0.86 across subscales (Kaufman, 2012).

#### Inventory of Creative Activities and Achievements (ICAA)

The ICAA (Diedrich et al., 2018) is a comprehensive evaluation of a person’s real-life engagement in eight domains of creativity: music, arts and crafts, creative cooking, sports, visual arts, performing arts and science and engineering. The ICAA has demonstrated strong internal consistency (α = 0.92; Diedrich et al., 2018). This self-report measure includes an assessment of both creative activities (little-C) and creative achievements (Pro-C).

##### Creative Activities subscale

The Creative Activities (CAct) subscale measures the frequency with which an individual has undertaken a given activity over the past 10 years. Each of the eight domains contained six items representing different activities. For example, in the literature domain, an item reads: “*Wrote a short literary work (e.g., poem, short story*)”. Responses were recorded on a 5-point Likert scale (0 = *never*; 1 = *1–2 times*; 2 = *3–5 times*; 3 = *6–10 times*; 4 = *more than 10 times*). A domain-general creative activities score was calculated by summing across the eight domains, with a maximum total possible score of 240. Higher scores indicate a higher frequency of creative activities. The CAct subscale demonstrates internal consistency ranging from α = 0.86 to 0.89 across studies, with test–retest reliability over a 4-week period at *r* = 0.81.

##### Creative Achievements subscale

The Creative Achievements (CAch) subscale measures participants’ creative achievements in each domain, ranging from personal engagement to public recognition. Unlike the CAct, which measures the frequency of creative engagement, the CAch subscale captures milestones in creative achievement. Participants check all levels of achievement they have attained on a 0–10 scale (0 = *“I have never been engaged in this domain”* to 10 = *“I have already sold some work in this domain*). The first five levels cover personal achievements (from *“I have tried this domain once”* to *“I have already taken classes to improve my skills”*), while the remaining levels cover public achievements (*“I have already published my original work in this domain”* to *“I have already sold work in this domain”*). Each checked levels of achievement contributes to the total score for that domain, with a maximum domain-specific score of 55. For example, in the literature domain, if the participant checks “1 = *I have tried this domain once*” and “2 = *I have already created at least one original work in this domain*”, their domain-specific score for literature would be 3. A domain-general creative achievements score was calculated by summing scores across all domains, with a maximum possible score of 440. Higher scores indicated greater levels of creative achievements. The CAch subscale also exhibits strong reliability (α = 0.88), though test–retest reliability is slightly lower (*r* = 0.67 over 4 weeks).

### Measures of creativity: Convergent and divergent thinking tasks

#### Convergent thinking: Remote Associates Test

To examine convergent thinking abilities, the Remote Associates Test (RAT; Mednick, 1962) was administered*.* Each trial consists of three cue words (e.g., *bald–screech–emblem*), each of which are linked to a target word (here: *eagle*) that participants must try to identify*.* The cue words were presented in the centre of the screen with a blank text box underneath, and participants were given 30 s to type their response in the box. Thirty trials were presented, 15 of which were taken from Mednick’s (1962) original items and 15 of which were developed for a previous study by our lab (see Open Science Framework: https://osf.io/ev7qm/?view_only=4109221c93264aa2ba63c885cf1b1ee3). Responses were corrected for spelling errors, and nonsensical words, incomplete responses, and timed-out responses were classified as inadequate responses and scored as incorrect. Responses that were very close synonyms to target words were accepted and marked as correct responses (e.g., *sea–ocean).* The final score for this task reflected the total number of correct responses. The original RAT demonstrates high internal reliability, with Spearman–Brown reliability coefficients reported as *r* = 0.92 and *r* = 0.91 in two separate samples (Mednick, 1962). Regarding validity, RAT scores correlate moderately to highly with measures of intelligence and problem-solving but more weakly with divergent thinking tasks, suggesting it primarily assesses convergent thinking processes (Lee et al., 2014; Mednick, 1962).

#### Divergent thinking: Alternate Uses Task

The Alternate Uses Task (Guilford, 1967) was used to assess divergent thinking. Participants were given the name of an everyday object and were asked to generate creative uses for the object in 2 min. The task was audio-recorded, and participants were asked to verbalize their responses out loud. Our main focus was the creative quality of the responses, so the participants were instructed to ‘*be as creative/original as possible*’, in line with recent recommendations regarding measurement and scoring of divergent thinking (Beaty et al., 2022; Reiter-Palmon et al., 2019). Accordingly, we explicitly instructed them to focus on two dimensions of creativity—namely, *originality* and *appropriateness*. Finally, they were told to prioritize quality over quantity via the following instruction: “*Come up with as many ideas as you can, but it’s more important to be creative than to come up with a lot of ideas*” (Nusbaum et al., 2014). Participants were given two cue words (*hanger, bucket*), and were given 2 minutes per item.

### Scoring the Alternate Uses Task

All responses were transcribed by the first author. For each item, responses were inspected for adequacy according to the guidelines in Guilford’s AUT test manual (Forthmann et al., 2016; Wilson et al., 1960). Accordingly, responses related to *selling, borrowing,* or *gifting* were marked as inadequate. Further, common uses for each object, repetitions, and vague, incomplete or nonsensical ideas were also excluded and marked as inadequate.

There are multiple methods for obtaining creativity metrics from the AUT, both empirical and subjective. However, there are concerns about discriminant validity amongst different methods of scoring the AUT—namely, fluency, flexibility, and originality (Acar et al., 2023; Benedek, 2024). A primary issue is the fluency confound, where higher response quantity (fluency) artificially inflates originality scores, making it difficult to determine whether individuals are truly generating more creative ideas or simply producing more responses overall. But these fluency-related biases can be mitigated using appropriate scoring methods. Given these considerations, we incorporated multiple creativity metrics—both empirical and subjective—to ensure a comprehensive evaluation that captures different facets of divergent thinking and minimizes potential biases associated with any single approach. Here, we use five of the most common measures: length of each response, fluency (number of responses), human ratings of creative quality, response rarity in the sample and AI-based scoring of response originality. We computed all five metrics to evaluate the extent to which they provided similar indices of divergent thinking, with higher scores signifying better performance.

#### Elaboration (response length)

Elaboration refers to the level of detail and explanation contained in each response (Guilford, 1967), indexed as the length of each response. We included this metric as automated methods of originality scoring may be biased by the number of words in a response. For example, responses with more words have lower LSA-derived semantic distance values (Forthmann et al., 2019a, 2019b, 2020a, 2020b). Moreover, human raters are also influenced by the specific way in which the idea is expressed and tend to rate longer responses as more creative (Beaty & Johnson, 2021). To account for this, elaboration of the response was scored using an unweighted word count approach using whitespace tokenization (i.e., counting the number of words in the response), which has been shown to be a reliable measure (Dumas et al., 2021).

#### Fluency (number of responses)

To index the productivity aspect of divergent thinking, fluency scores were computed for each item by summing up the total number of adequate responses and then averaging this across the two cue words.

#### Uniqueness (statistical rarity)

To index creative quality, we used an empirical measure of statistical rarity based on the frequency of the response in our sample (Forthmann et al., 2020a, 2020b). First, responses were categorized according to the degree of feature overlap, with functionally analogous uses grouped together (Reiter-Palmon et al., 2019). For example, possible uses of a bucket as a ‘shopping bag’ and ‘book bag’ would be grouped together as functioning to ‘*carry items*’. Next, a response occurrence table was built based on the number of equivalent responses that were identified and the frequency of each distinct response was estimated. An independent rater reviewed the categorization of a subset of the responses and expressed agreement with the coding scheme. Weights were assigned according to fixed threshold values of 1% and 5% (Runco, 2008). Responses that were provided by 1% of the sample were considered as *highly unique* and assigned a score of 2; those that were reported by less than 5% of the sample were considered *somewhat unique* and assigned a score of 1. All other responses were assigned a value of zero. Due to the potential confounding effects of fluency, Uniqueness scores for a participant’s responses were averaged rather than summed (Forthmann et al., 2020a, 2020b). A total uniqueness score for each participant was derived averaging the scores across responses for each cue and then averaging across the two cues.

#### Subjective creativity ratings (human ratings)

To measure the creative quality of responses, human ratings of creativity were obtained from 80 native English-speaking individuals with no prior experience. They were given some background information about the AUT and told that they would be asked to rate responses generated by participants from a previous study. Each response would be rated on its creative quality, on a scale of 1 (*not at all creative*) to 5 (*highly creative*)*.* The raters were provided with a set of instructions adapted from previous work (Silvia et al., 2008) that highlighted key dimensions of creative output: uncommonness, remoteness, and cleverness. Further, raters were told that strength in one of these dimensions could balance weakness in another dimension (e.g., a relatively common use that is expressed in a unique way could receive a higher score). The full instructions are provided in the Supplementary Materials (see Appendix A). Raters undertook some practice trials before they started with the main task. For each AUT cue word (i.e., *hanger* and *bucket*), equivalent responses and repetitions were grouped together (e.g., ‘as a plant pot’ and ‘planter’), while responses that were unique in the way they were worded or expressed (e.g., if they were more descriptive) remained individual cue words. For each cue word, two subsets of responses per age group were constructed, with each subset containing approximately 175–200 responses. Each subset was rated by 10 individuals who took approximately 25 min to complete the ratings. To standardise how responses were scored across subsets, each rater’s scores were normalised using the midpoint of the Likert scale (i.e., 3). The total creativity rating for a given response across raters was computed as the average of these normalised scores.

#### Originality (AI-based scoring)

To compute a measure of creative quality, responses were scored for originality using OCSAI (Open Creativity Scoring with Artificial Intelligence), an open-source software developed by Organisciak et al. (2022). This tool uses supervised machine learning to score AUT responses. It was developed by fine-tuning a neural network-based large-language model constructed with GPT-3 architecture (Brown, 2020), training it on past responses to the AUT. Across a large collection of datasets and prompts, Organisciak et al. (2022) found their model to be extremely strong at predicting human ratings of creativity (*r* = 0.81). Using the model, each response was scored on a scale of 1–5, where 1 was *very unoriginal* and 5 was *very original*. An overall originality score was calculated by averaging ratings across responses for each cue and then averaging across the two cues.

### Statistical analyses

First, to explore the relationships between the cognitive abilities, we computed Pearson’s correlations. The data were *z*-scored within each group prior to calculating the correlations to remove age group effects. Next, to investigate the separability of the divergent and convergent thinking constructs, we performed a principal component analysis of the creativity metrics. Components with eigenvalues greater than 1 were extracted and varimax rotated.

To examine whether semantic knowledge, semantic control, and executive abilities contribute to age differences in convergent thinking, and whether these predictors varied by age group, multilevel logistic regression models were run on RAT accuracy scores. Sum to zero coding was adopted for the Group factor, with older adults as the first level. To account for individual and trial-level variation, these models included random by-participant and by-trial intercepts. The model-building approach was as follows: our base model included *group*, *semantic knowledge*, *controlled retrieval*, and *semantic selection* as predictors, and in a second step, our full model also included measures of executive functioning (*updating, inhibition, shifting*) and broad cognitive ability (*speed of reasoning, fluid intelligence*). We also tested whether the effects of cognitive predictors differed between age groups. To do this, we iteratively compared the full model with models that included an interaction between group and each of the individual predictors using Likelihood Ratio Tests. If the addition of a group-level interaction term improved model fit, it was included in the final model. Continuous predictors were standardized prior to their inclusion in the model. Models were fit using maximum likelihood estimation and the “bobyqa” optimiser from the *lme4* package (Bates et al., 2015). *P* values were calculated using the *lmerTest* package (Kuznetsova et al., 2017), in which the degrees of freedom are approximated using Satterthwaite’s method.

Multiple regression models were run to investigate the contribution of semantic and executive abilities to divergent thinking performance, as indexed through our AUT metrics (*elaboration*, *fluency*, *uniqueness*, *human ratings*, and *originality*), and whether this varied by age group. Given that there were only two AUT trials, multiple regression was chosen over mixed-effects models, as there were not enough observations to estimate random effects. Initially, two models were run for each of the AUT outcomes: in the base model, the AUT metric was regressed on age group and semantic abilities; the full model additionally included all executive and broad cognitive functions as predictors. Elaboration has been established as an important confound in AUT scoring (Beaty & Johnson, 2021; Forthmann et al., 2019a, 2019b) and so was included as a covariate in all models. Following this, the incremental *F* tests were used to iteratively compare the full model to models with an interaction between age group and each of the individual predictors. If the group-level interaction term improved model fit, it was retained in the final model. Finally, if there was a significant group-level interaction, these effects were followed up with separate models in each age group, examining the effects of the semantic knowledge and control abilities, as well as executive abilities on each AUT metric.

## Results

### Age differences on measures of divergent thinking, semantic knowledge, and control mechanisms

The means and standard deviations of the variables of interest are shown in Table 1. Younger adults outperformed their older counterparts on tests of broad cognitive ability and executive functioning, including speed of reasoning, fluid intelligence, inhibition, switching, and updating. On the other hand, older adults showed superior performance on three out of four measures of semantic processing, including tests of semantic knowledge and controlled retrieval. There was no significant difference between groups on semantic selection.

**Table 1:** Descriptive statistics by age group

Ability	Task	Units	Younger adults	Older adults	Direction
Fluid intelligence	Series Completion	Total correct (max = 13)	7.35 (1.69)	6.25 (1.80)***	YA > OA
Speed of reasoning	Digit Symbol	Total correct in 90 s (max = 90)	49.52 (3.68)	37.62 (5.29)***	YA > OA
Inhibition	Colour-Word Interference	Time to completion (s)	44.17 (5.90)	56.87 (11.46)***	OA > YA
Updating	*N*-back task	Proportion Correct	0.94 (0.04)	0.88 (0.09)***	YA > OA
Shifting	Number–Letter Task	RT switch minus RT no switch (ms)	812.60 (482.07)	1,294.15 (786.47)***	YA > OA
Knowledge	Lexical Decision	% correct	78.94 (7.67)	93.94 (5.06)***	OA > YA
Knowledge	Synonym Judgement	% correct	37.5 (11.74)	71.55 (12.59)***	OA > YA
Knowledge	Lexical decision & synonym judgement	% correct	58.22 (8.37)	82.75 (8.14)***	OA > YA
Controlled retrieval	Weak Associates	Proportion correct (low association trials)	0.84 (0.07)	0.91 (0.06)***	OA > YA
Semantic selection	Incongruent Feature Selection	Proportion correct (incongruent trials)	0.82 (0.13)	0.85 (0.12)	ns
Convergent thinking	Remote Associates Test	Total correct (max = 30)	3.58 (0.13)	6.87 (2.38)***	OA > YA
DT elaboration	Alternate Uses Test	No. of words	9.22 (3.16)	10.35 (3.72)	ns
DT fluency	Alternate Uses Test	No. of responses (2 min)	7.43 (2.99)	9.63 (3.75)***	OA > YA
DT uniqueness	Alternate Uses Test	Uncommonness of responses (0–2)	0.47 (0.23)	0.46 (0.24)	ns
DT ratings	Alternate Uses Test	Subjective ratings (1–5 scale)	3.48 (0.11)	3.52 (0.12)	ns
DT originality	Alternate Uses Test	AI-based scoring (1–5 scale)	2.75 (0.19)	2.7*9* (0.19)	ns
Creative self-beliefs	K-DOCS	Self-report (range 50–250)	147.22 (21.75)	145.76 (15.05)	ns
Creative activities	ICAA	Self-report (max = 240)	65.70 (24.17)	78.37 (17.50)***	OA > YA
Creative achievements	ICAA	Self-report (max = 440)	71.22 (47.83)	27.97 (20.49)***	YA > OA

Mean (*M*) and standard deviation (*SD*) values are reported for each group. DT = Divergent Thinking; K-DOCS = Kaufman Domains of Creativity; ICAA = Inventory of Creative Activities and Achievements, Fluency = Number of responses; Uniqueness = Response rarity; Ratings = Human ratings; Originality = AI-based scoring; Elaboration = Word count. Standard deviations are shown in parentheses. Asterisks indicate the significance of *t* tests (uncorrected) comparing younger and older adults (older adults column). *p < 0.05; **p < 0.01; ***p < 0.001. ns = not significant

For creativity tasks, the older group demonstrated better convergent thinking than the younger group, with higher scores on the RAT. However, with respect to divergent thinking, there were no differences between the two groups on four out of five AUT metrics, including human ratings, uniqueness and originality. However, older adults were more fluent on the AUT, producing more responses than the younger group. Regarding self-report measures of creativity, older adults reported engaging in more creative activities, whereas the younger group reported more creative achievements. There was no difference in creative self-beliefs between groups. In summary, while the younger group exhibited superior broad cognitive and executive functioning abilities, the older group demonstrated advantages on tasks assessing semantic knowledge, controlled retrieval, and convergent thinking.

### Relationships between creativity and semantic and executive abilities

**Fig. 1:**
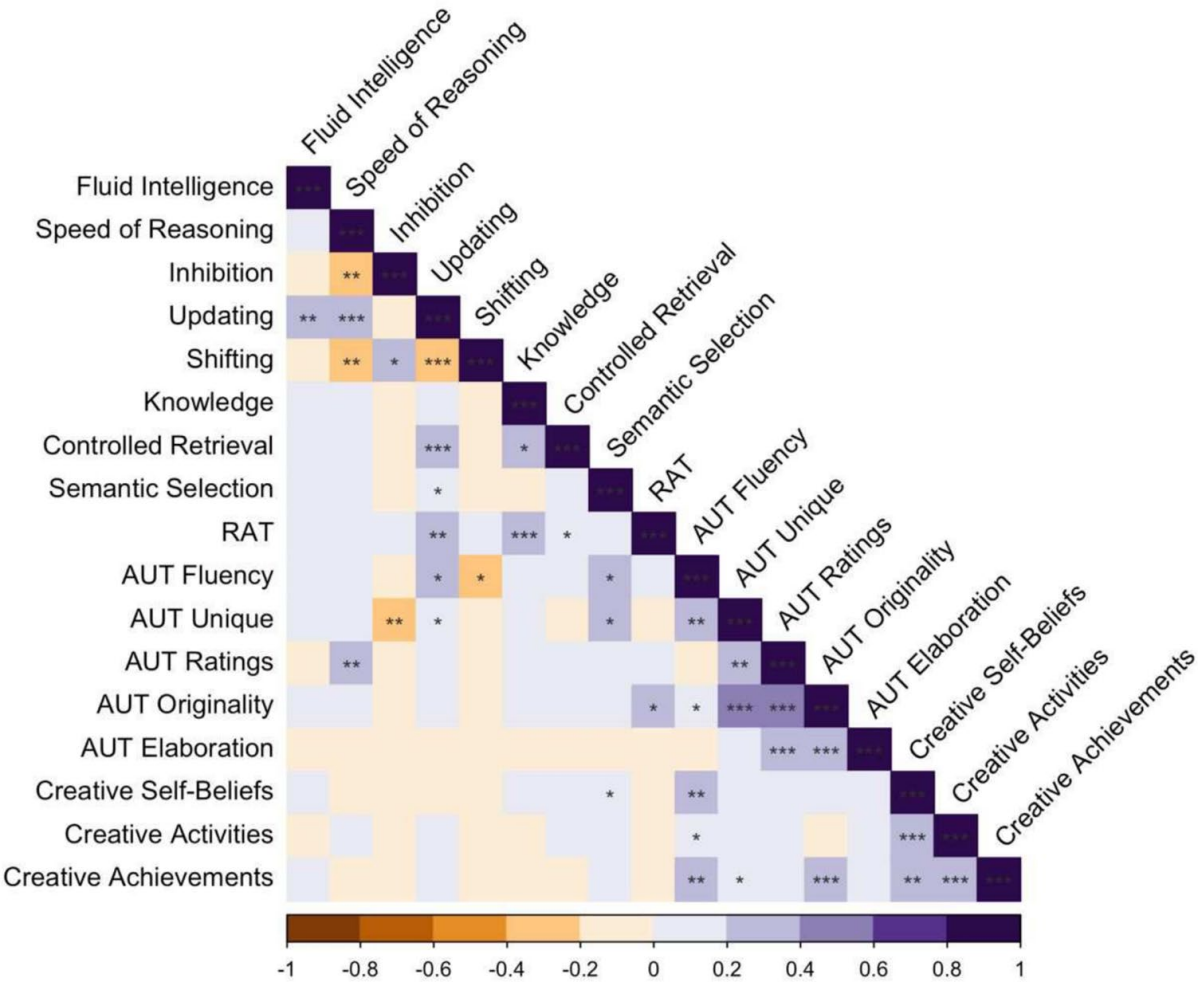
Pearson correlations between creativity, semantic knowledge and control mechanisms, and broad cognitive and executive control abilities in the combined dataset. *Note.* Inhibition and Shifting used time-based measures so higher values indicate poorer performance (unlike the other measures). RAT = Remote Associates Test; AUT = Alternate Uses Test; Fluency = Number of responses; Unique = Response rarity; Ratings = Human ratings; Originality = AI-based scoring; Elaboration = Word count. *p < 0.05; **p < 0.01; ***p < 0.001. (Colour figure online)

Pearson’s correlations were used to investigate the relationships between creativity and semantic and executive abilities. Figure 1 shows the relationships between measures in the full dataset. Scores were normalized within each group prior to computing correlations, so these effects are independent of age group differences.

The correlational analysis revealed distinct patterns of association amongst the broad cognitive abilities, executive functions, semantic processes, and creativity measures. Among the semantic measures, semantic knowledge and controlled retrieval correlated with one another but not with semantic selection, aligning with previous studies suggesting semantic selection is a distinct process from other aspects of semantic cognition (see Hoffman, 2018; Wu & Hoffman, 2022).

Examining the executive functions, both semantic control measures were associated with updating, but not with other executive and general cognitive measures, emphasising the distinction between these control processes. Among the core executive control measures, shifting was related to both inhibition and updating, but updating and inhibition were not related. Additionally, speed of reasoning correlated with all three core executive functions, while fluid intelligence showed a strong relationship to updating. Neither fluid intelligence nor speed of reasoning correlated with any of the semantic measures.

Divergent and convergent thinking seemed to be distinct constructs, with the RAT only weakly correlating with AUT originality and not any other AUT measures. Moreover, different sets of cognitive abilities seem to be related to divergent and convergent thinking. Across age groups, the RAT was correlated with updating, semantic knowledge, and controlled retrieval, implying that there may be some specific contributions of both semantic knowledge and control abilities, as well as executive control, to convergent thinking. On the other hand, divergent thinking presented a more complicated picture. While the different methods of scoring the AUT were strongly intercorrelated, there was a complex pattern of correlations between divergent thinking measures and the cognitive abilities, indicating that distinct executive and semantic control abilities are associated with aspects of divergent thinking. Additionally, while many of the associations were consistent across age groups, there also appeared to be some striking differences between age groups (separate correlational analyses presented in Appendix A, Fig. S1). First, while AUT uniqueness and fluency were correlated with semantic selection, this was primarily driven by the younger group. In contrast, correlations of AUT measures with broad cognitive abilities and executive functions—such as AUT ratings with speed of reasoning, and AUT uniqueness with inhibition and updating—were driven by the older group. However, there were some common trends across age groups. The relationship between AUT fluency with updating and shifting was present when both groups are combined, but did not reach significance in either group separately, suggesting the presence of some shared mechanisms. Together, these findings suggest that some of the cognitive mechanisms supporting divergent thinking may change with age, with younger adults relying more on semantic control and older adults relying on executive functions.

Finally, the self-report measures of creativity were correlated with one another, but were not associated with cognitive abilities, executive functions, semantic knowledge, or control abilities. The exception was one weak but significant correlation between creative self-beliefs and semantic selection. All three self-report measures were correlated with AUT fluency, but only creative self-achievements were correlated with AUT originality.

Taken together, these correlational findings demonstrate age-related differences in how cognitive abilities relate to creativity, suggesting that distinct mechanisms may drive creative thinking across the lifespan.

### Separability of divergent and convergent thinking

To explore the separability of divergent and convergent thinking, we performed an exploratory principal component analysis on the *z*-scored data. The Minimum Average Partial (MAP) method and Parallel Analysis suggested that a two-factor solution provided the best fit for the data, with the two components together accounting for approximately 46% of the variance in scores in each of the age groups (see Table 2). The first component appeared to index creative quality, as AUT metrics of uniqueness, human creativity ratings, originality, and elaboration all loaded on this factor. The second component appeared to index creative engagement, as it loaded on the self-report measures of creative behaviours and achievements, as well as the number of responses given in the AUT (Fluency). On the other hand, the RAT did not load strongly on either of these components, suggesting it may tap into a distinct dimension of creative thinking. To examine this further, we conducted regression models to understand which tests independently predicted these scores, when controlling for shared variance, and whether these relationships varied between age groups.

**Table 2:** Principal component analysis of creativity measures

	Component 1	Component 2
RAT	0.20	− 0.26
AUT Fluency	0.05	**0.62**
AUT Uniqueness	**0.57**	0.26
AUT Ratings	**0.78**	− 0.09
AUT Originality	**0.87**	0.10
AUT Elaboration	**0.60**	− 0.04
Creative Self-Beliefs	0.05	**0.68**
Creative Activities	− 0.05	**0.71**
Creative Achievements	0.30	**0.66**

RAT = Remote Associates Test; AUT = Alternate Uses Task; Fluency = Number of responses; Unique = Response rarity; Ratings = Human ratings; Originality = AI-based scoring; Elaboration = Word count;. Factor loadings > 0.4 are shown in bold

### Predictors of convergent thinking

Table 3 shows the effects in mixed models predicting RAT scores. In the first model, including semantic knowledge and control abilities as predictors, there was a strong effect of knowledge but no independent effects of controlled retrieval or semantic selection. Executive functions and general cognitive abilities were added in the second model, which significantly improved the model fit, as demonstrated by a likelihood ratio test, χ^2^(5) = 11.81, *p* < 0.05. The effect of knowledge remained highly significant in the second model: after controlling for executive abilities, the odds of correctly answering RAT items increased by 1.55 with every 1 standard deviation increase in semantic knowledge. Updating was also a significant predictor: better *N*-back performance predicted higher RAT scores, with a 1.19 increase in RAT performance for every 1 standard deviation increase in updating abilities. Effects of age group were not significant in either model, suggesting that older people’s advantage on the RAT is largely attributable to their more developed semantic knowledge. Finally, we examined whether any of the effects of cognitive scores varied by age group, but test x group interaction terms did not improve the model fit.

**Table 3:** Effects of age and semantic and executive functions on Remote Associates Test (RAT) performance

Effect	Model 1			Model 2		
	*OR*	CI	*p*	*OR*	CI	*p*
Group	1.17	0.96, 1.43	0.130	1.18	0.92, 1.52	0.184
Knowledge	1.49	1.21, 1.83	** < 0.001**	1.55	1.27, 1.90	** < 0.001**
Controlled Retrieval	1.09	0.95, 1.25	0.203	1.04	0.91, 1.19	0.584
Semantic Selection	1.02	0.91, 1.14	0.766	1.01	0.90, 1.12	0.904
Updating				1.19	1.04, 1.37	**0.013**
Inhibition				1.09	0.95, 1.25	0.197
Shifting				1.10	0.97, 1.24	0.140
Fluid intelligence				1.09	0.97, 1.23	0.148
Speed of reasoning				1.01	0.82, 1.25	0.892

### Predictors of divergent thinking

Table 4 shows effects in models predicting AUT *Elaboration* scores. Semantic knowledge and control abilities did not predict elaboration, indicating that these core semantic abilities may not drive the degree of elaboration in participants’ responses. Inhibition significantly predicted elaboration (β = − 0.88, 95% CI [− 1.73, − 0.03], *p* = 0.042), suggesting that a higher inhibition cost is associated with less elaborate responses.

**Table 4:** Analyses of effects of age, semantic and executive functions on Alternate Uses Task (AUT) Elaboration

Effect	Model 1				Model 2				
	*B* (*SE*)	CI	*p*		*B* (*SE*)		CI		*p*
Group	0.72	− 0.43, 1.86	0.217		1.04		− 0.45, 2.53		0.170
Semantic selection	− 0.07	− 0.71, 0.56	0.817		− 0.17		− 0.82, 0.48		0.605
Knowledge	0.05	− 1.11, 1.22	0.926		− 0.26		− 1.45, 0.93		0.666
Controlled retrieval	− 0.38	− 1.13, 0.36	0.310		− 0.29		− 1.07, 0.49		0.463
Updating					− 0.49		− 1.33, 0.35		0.248
Inhibition					− 0.88		− 1.73, − 0.03		**0.042**
Shifting					− 0.21		− 0.95, 0.53		0.572
Fluid intelligence					− 0.15		− 0.84, 0.54		0.667
Reasoning speed					− 0.31		− 1.52, 0.91		0.619
Group × Knowledge					− 1.15		− 2.30, 0.01		0.052

Elaboration = Length of the response

Table 5 shows effects in models predicting AUT *Fluency* scores. While the first model indicated that semantic selection was a significant predictor of Fluency, this effect was no longer present when accounting for executive and general cognitive functions in the final model. However, in the final model, there was a main effect of age group.

**Table 5:** Analyses of effects of age, semantic and executive functions on Alternate Uses Task (AUT) Fluency

Effect	Model 1				Model 2				
	*B* (*SE*)	CI	*p*		*B* (*SE*)		CI		*p*
Group	0.87	− 0.24, 1.98	0.122		1.56		0.11, 3.01		**0.035**
Semantic selection	0.67	0.06, 1.28	**0.032**		0.46		− 0.16, 1.07		0.145
Knowledge	− 0.02	− 1.14, 1.10	0.969		− 0.27		− 1.40, 0.86		0.632
Controlled retrieval	0.42	− 0.30, 1.14	0.247		0.41		− 0.34, 1.15		0.285
AUT elaboration	− 0.25	− 0.87, 0.37	0.420		− 0.16		− 0.79, 0.46		0.605
Fluid intelligence					0.39		− 0.26, 1.05		0.235
Reasoning speed					− 0.08		− 1.29, 1.14		0.898
Updating					0.30		− 0.50, 1.10		0.459
Inhibition					− 0.32		− 1.12, 0.49		0.438
Shifting					− 0.30		− 1.01, 0.40		0.396
Group × Fluid Intelligence					0.64		− 0.05, 1.34		0.069

AUT Fluency = Number of responses produced

Table 6 displays the effects in models predicting AUT *Uniqueness* scores. The first model indicated that semantic selection was a significant predictor of uniqueness. However, this effect disappeared when accounting for executive and general cognitive abilities. In the final model, there were no main effects or interactions with age group. Group level results are presented in Appendix A (Table S3).

**Table 6:** Analyses of effects of age, semantic and executive functions on Alternate Uses Task (AUT) Uniqueness

Effect	Model 1			Model 2				
	*B* (*SE*)	CI	*p*	*B* (*SE*)		CI		*p*
Group	− 0.04	− 0.12, 0.04	0.300	0.03		− 0.07, 0.12		0.616
Semantic selection	0.05	0.01, 0.10	**0.016**	0.04		− 0.00, 0.08		0.080
Knowledge	0.04	− 0.04, 0.11	0.364	0.02		− 0.06, 0.10		0.554
Controlled retrieval	− 0.01	− 0.06, 0.04	0.757	− 0.02		− 0.07, 0.04		0.517
AUT Elaboration	0.03	− 0.01, 0.08	0.118	0.03		− 0.01, 0.08		0.128
Updating				0.04		− 0.01, 0.10		0.150
Inhibition				− 0.05		− 0.10, 0.01		0.088
Shifting				− 0.01		− 0.06, 0.04		0.653
Fluid intelligence				− 0.00		− 0.05, 0.04		0.945
Reasoning speed				0.00		− 0.08, 0.08		0.957
Group × Semantic Selection				− 0.03		− 0.08, 0.01		0.113
Group × Controlled Retrieval				0.04		− 0.01, 0.09		0.141

AUT Uniqueness = Response rarity in study sample

**Fig. 2:**
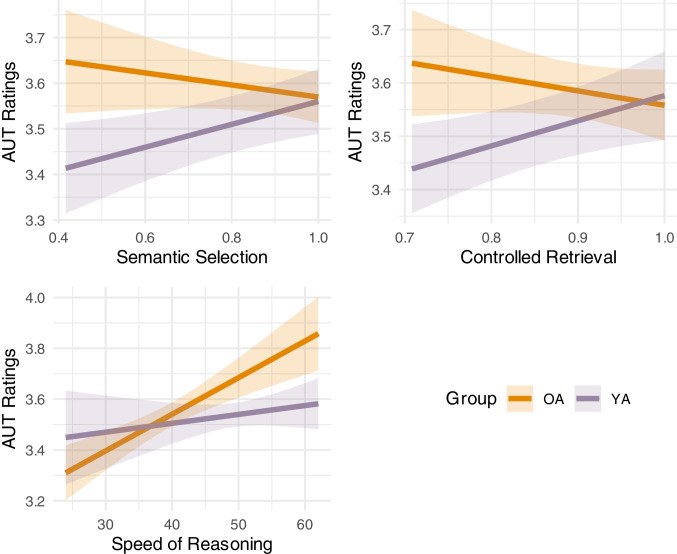
Effects of cognitive abilities on AUT Ratings. *Note.* OA = Older adults; YA = Younger adults; AUT Ratings = Human ratings of creativity (1–5 scale). The shaded areas represent the 95% confidence intervals. (Colour figure online)

Table 7 shows effects in models predicting AUT *Ratings* scores. In the first model, none of the semantic predictors had an effect on human ratings of creativity, but AUT elaboration was a significant covariate. The inclusion of executive functions, general cognitive abilities and interaction terms in the final model improved model fit, as demonstrated by an incremental *F* test, *F*(9) = 4.80, *p* < 0.001. In the final model, after controlling for executive abilities, there were significant interactions between group and semantic selection, controlled retrieval and speed of reasoning. Further, elaboration remained a significant covariate and there was a main effect of inhibition, where reduced inhibition predicted responses being rated as more creative. These interactions are shown in Fig. 2 and were investigated further in separate group level models. For younger adults, after controlling for executive abilities, human ratings of creative quality were predicted by semantic selection (β = 0.03, 95% CI [0.01, 0.06], *p* = 0.005) and controlled retrieval abilities (β = 0.04, 95% CI [0.01, 0.06], *p* = 0.004), but not speed of reasoning (β = 0.01, 95% CI [− 0.01, 0.04], *p* = 0.31), or inhibition (β = 0.01, 95% CI [− 0.02, 0.03], *p* = 0.50; See Appendix A, Table S2). However, this was not the case for the older group, wherein human ratings of creativity were positively predicted by speed of reasoning (β = 0.07, 95% CI [0.03, 0.11], *p* = 0.001), and interestingly, by inhibition (β = 0.04, 95% CI [0.00, 0.07], *p* = 0.032), suggesting that reduced inhibitory control may be related to greater creativity in older adults. In contrast, semantic selection (β = − 0.02, 95% CI [− 0.05, 0.01], *p* = 0.283) and controlled retrieval (β = − 0.02, 95% CI [− 0.05, 0.01], *p* = 0.185) did not predict creativity in this group.

**Table 7:** Analyses of effects of age, semantic and executive functions on Alternate Uses Task (AUT) Ratings

Effect	Model 1			Model 2		
	*B* (*SE*)	CI	*p*	*B* (*SE*)	CI	*p*
Group	0.00	− 0.03, 0.04	0.891	0.04	− 0.00, 0.08	0.075
Semantic selection	0.01	− 0.01, 0.03	0.295	0.01	− 0.01, 0.03	0.454
Knowledge	0.00	− 0.03, 0.04	0.864	− 0.00	− 0.03, 0.03	0.980
Controlled retrieval	0.01	− 0.01, 0.03	0.356	0.01	− 0.01, 0.03	0.503
AUT Elaboration	0.04	0.02, 0.06	** < 0.001**	0.05	0.03, 0.07	** < 0.001**
Reasoning speed				0.07	0.03, 0.10	** < 0.001**
Updating				− 0.00	− 0.03, 0.02	0.877
Inhibition				0.03	0.00, 0.05	**0.030**
Shifting				− 0.00	− 0.02, 0.02	0.948
Fluid intelligence				− 0.01	− 0.03, 0.01	0.272
Group × Semantic Selection				− 0.02	− 0.04, − 0.01	**0.008**
Group × Controlled Retrieval				− 0.03	− 0.05, − 0.01	**0.011**
Group × Reasoning Speed				0.04	0.01, 0.07	**0.023**
Group × AUT Elaboration				− 0.02	− 0.04, 0.00	0.067

AUT Ratings = Human ratings of creativity (1–5 scale)

**Fig. 3:**
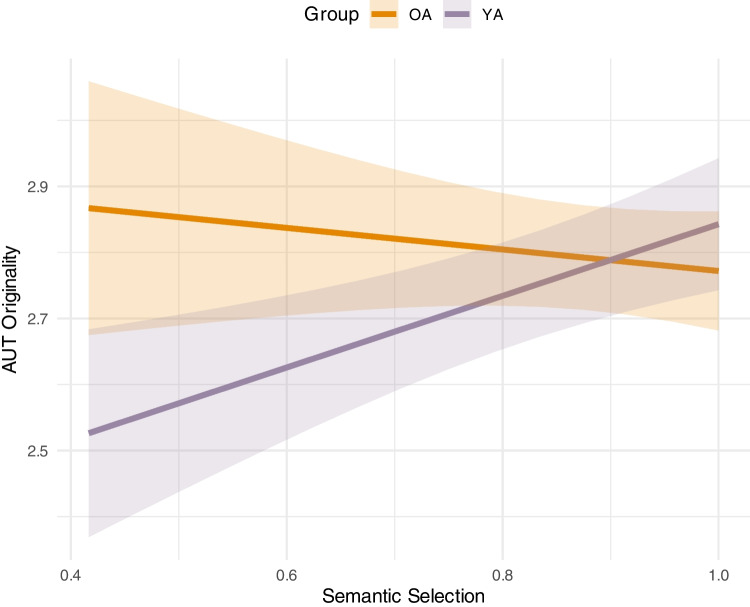
Two-way interaction plot of the predicted effects of semantic selection on AUT Originality scores. *Note.* OA = Older adults; YA = Younger adults; AUT Originality = Scores derived using AI-based ratings (1–5 scale). The shaded areas represent the 95% confidence intervals. (Colour figure online)

Table 8 demonstrates the effects in regression models predicting AUT *Originality* scores. The first model included only the semantic predictors, none of which significantly predicted originality. AUT elaboration was also included as a covariate in all AUT models, and as expected, more elaborate responses received higher originality scores. The final model included executive functions, general cognitive abilities and interactions of abilities with age group (wherever these improved model fit). The final model was a better fit for the data, as demonstrated by an incremental *F* test, *F*(7) = 2.40, *p* = 0.024. There were no main effects of test scores but there was a significant interaction between group and semantic selection, and elaboration remained a significant covariate. The interaction effect is shown in Fig. 3. We investigated this interaction by estimating separate models for each age group (see Table 1 in Appendix C). There were no semantic control effects in the older group; however, in the younger group, after controlling for executive abilities, participants with better semantic selection ability produced more original AUT responses (β = 0.07, 95% CI [0.02, 0.11], *p* < 0.01). Group-level results are presented in Appendix A (Table S1).

**Table 8:** Analyses of effects of age, semantic and executive functions on the Alternate Uses Task (AUT) Originality

Effect	Model 1			Model 2				
	*B* (*SE*)	CI	*p*	*B* (*SE*)		CI		*p*
Group	− 0.02	− 0.08, 0.04	0.470	0.03		− 0.04, 0.10		0.440
Semantic selection	0.03	− 0.00, 0.06	0.057	0.02		− 0.01, 0.05		0.150
Knowledge	0.02	− 0.04, 0.07	0.565	0.01		− 0.05, 0.07		0.705
Controlled retrieval	0.02	− 0.02, 0.06	0.311	0.01		− 0.03, 0.05		0.568
AUT Elaboration	0.07	0.04, 0.11	** < 0.001**	0.08		0.05, 0.11		** < 0.001**
Updating				0.02		− 0.02, 0.06		0.304
Inhibition				0.01		− 0.03, 0.05		0.684
Shifting				0.02		− 0.02, 0.05		0.298
Fluid intelligence				0.03		− 0.01, 0.06		0.140
Reasoning speed				0.05		− 0.01, 0.10		0.126
Group × Semantic Selection				− 0.04		− 0.07, − 0.01		**0.006**
Group × Controlled Retrieval				− 0.03		− 0.06, 0.01		0.157

AUT Originality = Scores derived using AI-based ratings (1–5 scale)

## Discussion

The present study sought to understand age-related changes in divergent and convergent thinking, and the specific contributions of semantic knowledge and control as well as executive abilities to creative thought in younger and older adults. While older adults showed superior convergent thinking abilities, both groups displayed comparable divergent thinking performance across several metrics of creative quality, including AI-based originality scoring, human ratings of creativity and statistical uncommonness of responses. Importantly, our work puts forth two interesting findings: first, divergent and convergent thinking emerged as distinct constructs relying on different sets of cognitive abilities, and second, there were age-related differences in the recruitment of these abilities for creative pursuits.

### Age-related differences in cognitive abilities

Older adults exhibited deficits in executive functioning, consistent with previous work (Argiris et al., 2020; Bopp & Verhaeghen, 2018; Idowu & Szameitat, 2023; MacPherson et al., 2002; Maldonado et al., 2020; Rey-Mermet & Gade, 2018; Salthouse, 2009; Verhaeghen & Salthouse, 1997; Wasylyshyn et al., 2011). However, they also exhibited preserved semantic knowledge, in line with numerous studies chronicling increases in semantic knowledge with age, accrued through years of education and lifelong literary, cultural and workplace experience (Hoffman, 2018; Verhaeghen, 2003). Interestingly, while older adults exhibited a greater ability to engage in the controlled retrieval of less salient semantic associations, this did not extend to their performance on the semantic selection task, replicating previously established age-group dissociations between these two aspects of semantic control (Hoffman, 2018; Hoffman & MacPherson, 2022). Notably, while our sample of older adults performed numerically more poorly on the semantic selection task, the difference between age groups was not statistically significant (unlike Hoffman, 2018). As our sample was younger than that of Hoffman (2018), it is possible that age differences in this task are more pronounced when much older participants are included.

In tests of creativity performance, the older group demonstrated superior convergent thinking skills, but with respect to divergent thinking, both younger and older people performed similarly on measures of creative quality indexed by originality, subjective ratings, and uniqueness. Notably, there were age differences in fluency and elaboration: older people generated more responses, and their responses tended to be longer. Our results align with literature suggesting that, despite declines in executive abilities, verbal divergent thinking abilities are preserved in older adults (Fusi et al., 2021; Madore et al., 2016). Additionally, we extend this work on creativity and aging by highlighting the potential for older adults to show enhanced convergent thinking abilities.

### Relationships between cognitive abilities and creative thinking

Across groups, breadth of semantic knowledge was correlated with controlled retrieval abilities, indicating that individuals with a larger base of semantic information develop more effective search mechanisms. Nevertheless, like Hoffman (2018) and Wu and Hoffman (2022), semantic selection was not related to the other semantic abilities, providing further support for the distinction between these two types of semantic control. Finally, both controlled retrieval and semantic selection performance were related to updating, demonstrating the importance of being able to hold and manipulate multiple pieces of information in mind when engaging in strategic semantic retrieval and selection between competing alternatives.

Building on the relationships between semantic and executive functions, our analysis also revealed how these cognitive abilities relate to divergent and convergent thinking, highlighting shared and distinct patterns of cognitive engagement for the two modes of thinking in younger and older adults. Across both age groups, the RAT was related to updating, semantic knowledge and controlled retrieval, suggesting that both semantic and executive abilities contribute to convergent thinking. However, divergent thinking, assessed by the AUT, exhibited a more complex pattern of associations: younger adults’ performance was more strongly related to semantic control, whereas older adults showed stronger links to general cognitive abilities (speed of reasoning) and executive functions (inhibition).

### Separability of divergent and convergent thinking

The literature has poised divergent and convergent thinking as distinct but interrelated elements of creativity, with inconsistent associations between the two (Lee & Therriault, 2013; Lee et al., 2014). This was supported in our data: While the RAT was weakly correlated to one measure of creative quality of divergent thinking responses (AI-based originality scores), a principal components analysis confirmed that the RAT and the AUT metrics tap into different cognitive constructs. Specifically, the principal components analysis suggested two separable factors: *creative quality*, capturing the uniqueness, originality, degree of elaboration, and subjective human ratings of AUT responses; and *creative engagement*, reflecting the quantity of ideas generated on the AUT, as well as self-reported creative beliefs, behaviours and achievements. Notably, the RAT did not load strongly on either component, reinforcing the idea that divergent and convergent thinking are distinct cognitive constructs. This is further supported by the results of our regression analyses that reveal how different cognitive abilities contribute to each mode of creative thinking.

### Predictors of convergent thinking

Our findings on convergent thinking align with the existing literature, implicating both semantic knowledge and executive functions in this process. Importantly, these trends were consistent across both younger and older adults, suggesting that the cognitive mechanisms underlying convergent thinking are similarly engaged by both age groups. In our study, people with larger stores of semantic knowledge and better executive updating abilities demonstrated better performance on the RAT, after accounting for semantic control and other executive abilities. Indeed, to solve a RAT item, individuals must hold multiple cues in mind while searching through the intersection of their semantic neighbourhoods for the related target word, following which, they engage in a sequential evaluation of candidate responses (Smith et al., 2013). Thus, greater reserves of conceptual knowledge may provide an individual with a larger search space for the bottom-up spreading of activation processes during the idea generation phase. This finding aligns with previous research indicating that crystallized intelligence, a measure that taps vocabulary and verbal intelligence, uniquely predicts multiply-constrained problem-solving tasks, including the RAT (Ellis et al., 2021).

Additionally, our findings suggest that semantic knowledge alone is not sufficient to solve RAT problems. Updating, the ability to manipulate the contents of working memory, was also integral to convergent thinking performance. This aligns with previous work that has highlighted the importance of working memory capacity for multiply constrained problem-solving tasks like the RAT (Ellis & Brewer, 2018; Ellis et al., 2021). This top-down executive control allows for the maintenance and manipulation of the three cues in working memory while searching through semantic memory and comparing each potential solution to the features of the cue words.

### Predictors of divergent thinking

Our findings indicate that while younger and older adults exhibit similar levels of divergent thinking, the cognitive mechanisms underlying this ability differ between the groups. In the younger group, AUT performance was driven by semantic control mechanisms: controlled retrieval predicted human ratings of creative quality and uniqueness; while semantic selection influenced originality, human ratings, and uniqueness. In contrast, for the older group, AUT performance was driven by the general cognitive ability of speed of reasoning, and the executive function of inhibition. Interestingly, our study did not find that traditional factors that are commonly associated with divergent thinking, like broad cognitive abilities of fluid intelligence and the other core executive functions, were significant predictors of performance. This suggests that these factors may not always play a central role in divergent thinking, especially when other cognitive resources are considered.

Semantic control may be one such factor that is particularly relevant for divergent thinking, although it has not been extensively studied in this context. Divergent thinking tasks, such as the AUT, requires the generation of multiple uncommon, but task-appropriate, ideas. When the passive spread of semantic activation does not result in novel ideas, *controlled retrieval* mechanisms could allow individuals to engage in a strategic search for more remote associations. This generative process may trigger the retrieval of multiple competing ideas, requiring *semantic selection* processes to evaluate potential solutions and filter out less promising ideas. Together, these processes could enable more efficient generation of original yet task-appropriate ideas. This pattern of recruitment suggests that younger people, with less knowledge and experience of the world, may rely on semantic control to leverage their limited knowledge and flexibly link disparate pieces of information.

On the other hand, semantic control mechanisms were less important for divergent thinking in older adults. This could be explained by the nature of the AUT itself, which engages both semantic and episodic associations (Ahmed et al., 2023; Benedek et al., 2023). When thinking of new uses for an object, individuals draw on object properties as well as personal memories of object use. In aging, episodic memory becomes increasingly ‘semanticized’ (i.e., detailed memories of personal events become integrated into broad semantic knowledge; Spreng & Turner, 2019). This may be particularly relevant in the context of the AUT, as the ideas generated may not be novel to the individual, even if those ideas are original or uncommon in the sample. Older adults may have leveraged their extensive conceptual knowledge and life experiences, retrieving strongly encoded episodic and semantic information more easily from their existing knowledge base without having to rely on semantic control. As a result, they can generate ideas more fluently, benefitting from their rich knowledge base, but may be constrained by more general cognitive factors like speed of reasoning. Interestingly, while age-related declines in inhibitory control are typically seen as a disadvantage, this may paradoxically support creativity in later life (Amer et al., 2022; Yang et al., 2022). In this context, weaker inhibition may reduce the filtering of more loosely related or unconventional associations, enabling older adults to access a more diverse pool of ideas. This wider search space could promote originality by allowing more unexpected connections to surface. Taken together, our results suggest that, while older and younger adults displayed equivalent divergent thinking abilities, these groups may differentially draw on cognitive processes to achieve the same outcome.

### Implications for cognitive aging

Our findings align with the default-executive coupling hypothesis of aging (DECHA; Spreng & Turner, 2019) which proposes that, with age, there is a shift toward semanticized cognition (i.e., an increased reliance on prior knowledge, including semantic, conceptual and schematic information), coupled with declines in cognitive control. The authors suggest that this reliance on prior knowledge leads to a shift in cognitive mode (Hills et al., 2015) from exploratory (novelty-seeking, preference for external environment) to exploitative (novelty-aversive, reliance on prior knowledge). At the neural level, this is reflected in adaptive functional changes in network connectivity. Aging is associated with greater coupling between the executive network (i.e., brain regions linked goal-directed behaviour and cognitive control) and default network (i.e., brain regions engaged during internally generated thought; Spreng & Turner, 2019). Supporting this view, Adnan et al. (2019) found that older adults exhibited more default-executive coupling that their younger counterparts, despite both groups performing similarly on a divergent thinking task. Critically, older adults who demonstrated greater network integration (i.e., less segregation between the default-executive regions) were also rated as being more creative. These results suggest that this network integration may be adaptive in aging, allowing older adults to offset losses in executive control by using their wealth and variety of experiences to maintain their creative abilities.

This framework provides a useful lens for interpreting our findings. In our study, the older group leveraged their rich semantic knowledge to solve RAT problems more effectively than younger adults, despite their declines in updating ability. However, unlike the RAT, simply having a larger store of knowledge did not confer any benefits to performance on the AUT. Convergent thinking tasks like the RAT have multiple cue constraints and require individuals to arrive at a specific target response. This cannot happen unless individuals already possess the specific information required (i.e., the relevant relationships between each of the cue words and the target), conferring an advantage on individuals who have a wider knowledge base. On the other hand, divergent thinking tasks are more flexible, open-ended, and can be approached in many ways. Here, what an individual *knows* may be less critical than how *efficiently* they can identify and use their knowledge in a novel way. Finally, it could also be that our knowledge measure did not capture the most relevant forms of knowledge for the AUT. We operationalized knowledge as verbal vocabulary knowledge—while this may be important for the RAT, it may not be as relevant for the AUT, which could depend instead on non-verbal object knowledge and general life experiences.

### Methodological considerations

One of the key methodological strengths of this study was our use of multiple metrics to assess divergent thinking, including human ratings of creative quality, AI-based scoring of originality, and a measure of statistical uncommonness in the sample. While all the AUT metrics were correlated, the strong association between the AI-based originality scoring and human ratings underscores the potential of automated scoring approaches as a viable alternative to the more time consuming, human-based scoring (Buczak et al., 2023; Organisciak et al., 2022). Moreover, the use of multiple metrics highlights a key discrepancy in our analysis: Fluency and uniqueness were significantly correlated with cognitive abilities, but our regression models only identified significant predictors for originality and human ratings. This suggests that while fluency and uniqueness may provide a broad measure of idea generation, the *quality* of those ideas—as captured by originality and human ratings—is more strongly influenced by underlying cognitive abilities. While uniqueness is useful proxy for originality, it is more crude measure of divergent thinking, requiring very large samples to accurately estimate the rarity of a response at the population level (Forthmann et al., 2020a, 2020b; Reiter-Palmon et al., 2019).

To conclude, the current study demonstrated that semantic knowledge contributes to convergent thinking in younger and older people, but that these groups may differentially recruit semantic and executive abilities for divergent thinking. These findings are in line with neurocognitive theories of aging. Moreover, our study provided further evidence that older people are as successful at divergent thinking tasks as their younger counterparts. While aging is related to cognitive decline across several areas, especially those related to cognitive control, age also brings a wealth of knowledge and life experience that helps older people manoeuvre through daily life. This knowledge seems to be especially relevant for older adults to engage in creative thinking to find new ways to adapt when navigating everyday problem-solving.

## Supplementary Information

Below is the link to the electronic supplementary material.

Supplementary file1 (DOCX 300 KB)

## Data Availability

Data and code for the study can be accessed online (https://osf.io/ev7qm/?view_only=4109221c93264aa2ba63c885cf1b1ee3), and study materials can be provided upon request.

## Code availability

See above.
