# Supplementary material for: Investigating age-related differences in semantic control mechanisms involved in creative cognition
Source: Mem Cognit. 2025 Jul 11;54(1):335–60. doi: 10.3758/s13421-025-01753-6 (PMC12864212; doi:10.3758/s13421-025-01753-6)
Supplement: Supplementary file 1 — Supplementary file1 (DOCX 300 KB) [file 13421_2025_1753_MOESM1_ESM.docx]

**Supplementary Materials**

Appendix A: Instructions for Creativity Ratings

Background

You are going to be presented with a series of original responses from a previous study on Creativity. Each of the responses was produced by participants while they were performed a classic creativity task called the ‘*Alternate Uses Task*’ (AUT). In the AUT, participants are presented with a common, everyday object *(e.g., ‘a rope’)* and asked to produce alternative uses for the object *(e.g., ‘pile of rope as a pillow’).*

What you will have to do

Your task is to read through each of the responses and rate each response on how creative it is.  You will be assigning each response a single score, using a scale of 1 (*Not at all Creative*) to 5 (*Highly Creative*).

When you are scoring, you need to keep in mind three dimensions of creative ideas.

1. **Uncommon**: Creative ideas are uncommon; they will occur infrequently in the sample of responses, and they will be unique. *Note, just because a response is only given once, it need not be judged as creative. For example, a random or inappropriate response would be uncommon but not creative.*
2. **Remoteness:** Creative ideas are remotely linked to everyday objects and ideas. For example, creative uses for a brick are far from the common uses for a break. Responses that stray from obvious ideas are more creative, and responses that are close to obvious ideas are uncreative.
3. **Clever:** Creative ideas are often clever; they strike people as insightful, ironic, humorous, fitting, or smart. Responses that are clever will tend to be creative responses. *Keep in mind that cleverness can compensate for other facets. For example, a common use that is cleverly expressed could receive a high score.*

1. To recap, when rating each item, think of the following dimensions of creativity: **uncommonness, remoteness, and cleverness.** Strength in one dimension can balance weakness in another.
2. You might come across some responses that have been repeated – try to mark all the responses of one kind in the same way. (Unless the response is *different* in some way from the other responses of the same kind, e.g., if it is more descriptive)
3. Give low scores to any responses that **list the actual uses of the object.** e.g., if the object was a brick, and the response is ‘*to make a wall*’ or ‘*to make a fireplace*’, then you would give these responses a score of 1 (*Not at all Creative*) as they are the actual uses of the brick.
4. Give low scores to any responses that are **incomplete, non-understandable, or nonsensical.** e.g., If the object was a brick, and the response was ‘*to eat*’, then you would give this response a score of 1 (*Not at all Creative*) as you cannot eat a brick.
5. Give low scores to vague ideas. e.g., if the idea is under-developed and you cannot form a full picture of the intended use with the information given.

**Appendix B: Correlations in each age group**


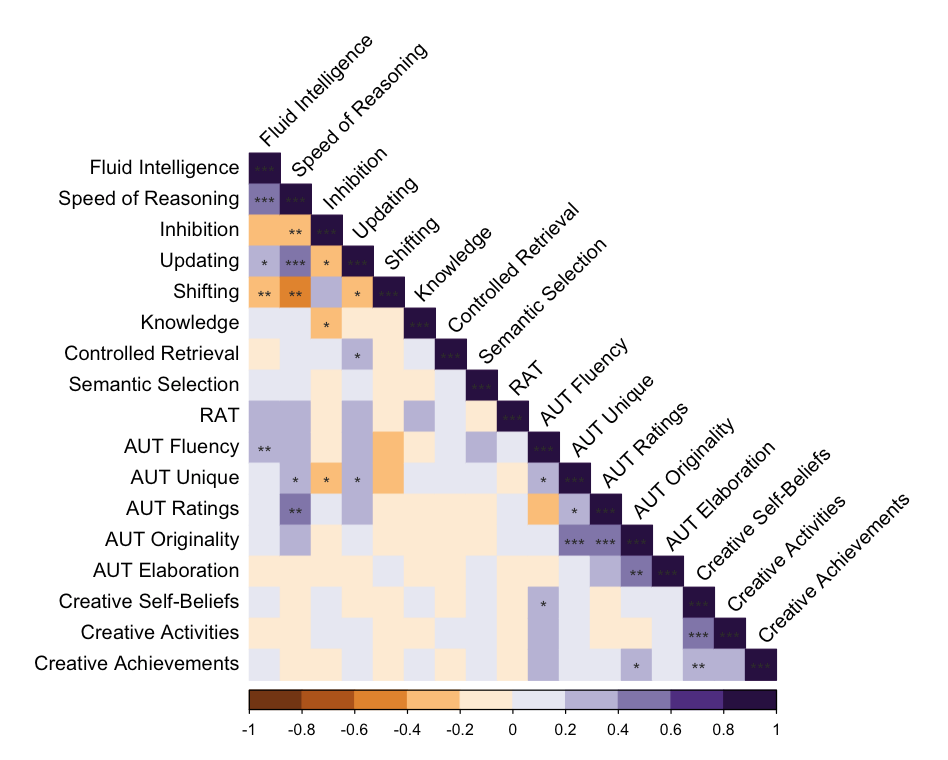


*Figure S1: Pearson correlations between semantic knowledge and control and executive abilities in the older group. Note that Inhibition and Shifting used time-based measures so higher values indicate poorer performance (unlike the other measures), RAT = Remote Associates Test; AUT = Alternate Uses Test.*

** = p < 0.05; ** = p < 0.01; *** = p < 0.001.*


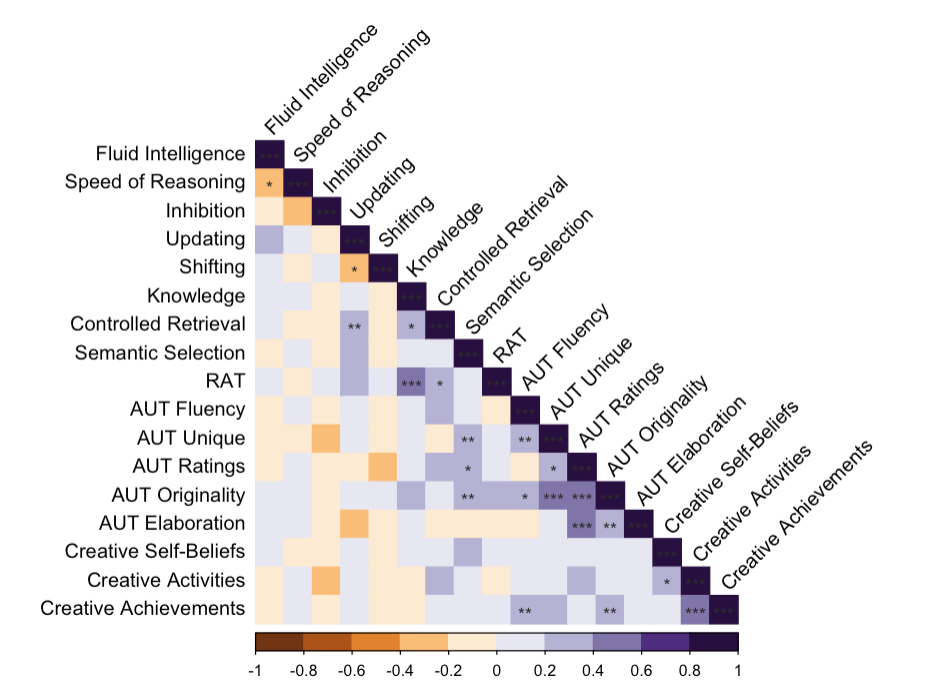


*Figure S2: Pearson correlations between semantic knowledge and control, and executive abilities in the younger group. Note that Inhibition and Shifting used time-based measures so higher values indicate poorer performance (unlike the other measures), RAT = Remote Associates Test; AUT = Alternate Uses Test.*

** = p < 0.05; ** = p < 0.01; *** = p < 0.001.*

**Appendix C: Group-level models**

*Table S1: Analysis of effects of semantic knowledge and control, and executive abilities on Alternate Uses Task (AUT) Originality within the older and younger groups.*

| Effect |  | Older Adults | |  | |  | | Younger Adults |  |
| --- | --- | --- | --- | --- | --- | --- | --- | --- | --- |
|  | B (se) | | CI | p | B (se) | | CI | | p |
| Semantic Selection | -0.02 | | -0.07 – 0.02 | 0.307 | 0.07 | | 0.02 – 0.11 | | **0.003** |
| Knowledge | -0.01 | | -0.06 – 0.05 | 0.841 | 0.02 | | -0.03 – 0.06 | | 0.477 |
| Controlled Retrieval | -0.01 | | -0.06 – 0.04 | 0.761 | 0.03 | | -0.02 – 0.08 | | 0.216 |
| AUT Elaboration | 0.09 | | 0.04 – 0.14 | **<0.001** | 0.08 | | 0.03 – 0.13 | | **0.001** |
| Updating | 0.02 | | -0.04 – 0.08 | 0.548 | 0.02 | | -0.03 – 0.07 | | 0.386 |
| Inhibition | 0.01 | | -0.04 – 0.06 | 0.707 | -0.00 | | -0.05 – 0.04 | | 0.894 |
| Shifting | 0.01 | | -0.04 – 0.06 | 0.636 | 0.04 | | -0.01 – 0.08 | | 0.120 |
| Fluid Intelligence | 0.04 | | -0.01 – 0.10 | 0.112 | -0.00 | | -0.05 – 0.05 | | 0.962 |
| Reasoning Speed | 0.03 | | -0.03 – 0.09 | 0.324 | 0.01 | | -0.04 – 0.05 | | 0.748 |

*Note: AUT Originality = Scores derived using AI-based ratings (1-5 scale).*

*Table S2: Analysis of effects of semantic knowledge and control, and executive abilities on Alternate Uses Task (AUT) Subjective Ratings within the older and younger groups.*

| Effect |  | Older Adults | |  | |  | | Younger Adults |  |
| --- | --- | --- | --- | --- | --- | --- | --- | --- | --- |
|  | B (se) | | CI | p | B (se) | | CI | | p |
| Semantic Selection | -0.02 | | -0.05 – 0.01 | 0.283 | 0.03 | | 0.01 – 0.06 | | **0.005** |
| Knowledge | 0.01 | | -0.02 – 0.04 | 0.517 | -0.01 | | -0.03 – 0.02 | | 0.559 |
| Controlled Retrieval | -0.02 | | -0.05 – 0.01 | 0.185 | 0.04 | | 0.01 – 0.06 | | **0.004** |
| AUT Elaboration | 0.04 | | 0.01 – 0.07 | **0.009** | 0.06 | | 0.03 – 0.08 | | **<0.001** |
| Updating | 0.01 | | -0.03 – 0.05 | 0.501 | -0.02 | | -0.05 – 0.00 | | 0.107 |
| Inhibition | 0.04 | | 0.00 – 0.07 | **0.032** | 0.01 | | -0.02 – 0.03 | | 0.505 |
| Shifting | 0.01 | | -0.03 – 0.04 | 0.709 | -0.01 | | -0.04 – 0.01 | | 0.259 |
| Fluid Intelligence | -0.01 | | -0.04 – 0.03 | 0.602 | -0.01 | | -0.03 – 0.02 | | 0.517 |
| Reasoning Speed | 0.07 | | 0.03 – 0.11 | **0.001** | 0.01 | | -0.01 – 0.04 | | 0.317 |
|  |  | |  |  |  | |  | |  |

*Note: AUT Ratings = Human ratings of creativity (1-5 scale).*

*Table S3: Analysis of effects of semantic knowledge and control, and executive abilities on Alternate Uses Task (AUT) Uniqueness within the older and younger groups.*

| Effect |  | Older Adults | |  | |  | | Younger Adults |  |
| --- | --- | --- | --- | --- | --- | --- | --- | --- | --- |
|  | B (se) | | CI | p | B (se) | | CI | | p |
| Semantic Selection | 0.00 | | -0.07 – 0.07 | 0.998 | 0.07 | | 0.01 – 0.13 | | **0.019** |
| Knowledge | -0.01 | | -0.08 – 0.06 | 0.871 | 0.04 | | -0.02 – 0.10 | | 0.208 |
| Controlled Retrieval | 0.02 | | -0.05 – 0.09 | 0.520 | -0.07 | | -0.13 – -0.01 | | **0.031** |
| AUT Elaboration | 0.04 | | -0.03 – 0.11 | 0.244 | 0.03 | | -0.03 – 0.09 | | 0.331 |
| Updating | 0.02 | | -0.06 – 0.11 | 0.619 | 0.05 | | -0.02 – 0.12 | | 0.145 |
| Inhibition | -0.04 | | -0.11 – 0.04 | 0.328 | -0.07 | | -0.13 – -0.01 | | **0.033** |
| Shifting | -0.02 | | -0.10 – 0.05 | 0.516 | 0.02 | | -0.04 – 0.09 | | 0.418 |
| Fluid Intelligence | 0.00 | | -0.07 – 0.08 | 0.959 | -0.03 | | -0.09 – 0.03 | | 0.364 |
| Reasoning Speed | 0.04 | | -0.05 – 0.12 | 0.400 | -0.04 | | -0.10 – 0.02 | | 0.158 |

*Note: AUT Uniqueness = Response rarity in study sample.*

*Table S4: Analysis of effects of semantic knowledge and control, and executive abilities on Alternate Uses Task (AUT) Fluency within the older and younger groups.*

| Effect |  | Older Adults | |  | |  | | Younger Adults |  |
| --- | --- | --- | --- | --- | --- | --- | --- | --- | --- |
|  | B (se) | | CI | p | B (se) | | CI | | p |
| Semantic Selection | 0.52 | | -0.48 – 1.52 | 0.301 | 0.32 | | -0.52 – 1.16 | | 0.449 |
| Knowledge | -0.40 | | -1.47 – 0.67 | 0.454 | 0.03 | | -0.83 – 0.89 | | 0.944 |
| Controlled Retrieval | 0.25 | | -0.80 – 1.31 | 0.633 | 0.41 | | -0.50 – 1.32 | | 0.365 |
| AUT Elaboration | -0.28 | | -1.29 – 0.72 | 0.573 | -0.09 | | -0.98 – 0.80 | | 0.845 |
| Updating | 0.26 | | -1.01 – 1.54 | 0.680 | 0.34 | | -0.63 – 1.30 | | 0.483 |
| Inhibition | -0.39 | | -1.51 – 0.73 | 0.484 | -0.20 | | -1.07 – 0.68 | | 0.654 |
| Shifting | -0.29 | | -1.38 – 0.80 | 0.594 | -0.27 | | -1.14 – 0.61 | | 0.547 |
| Fluid Intelligence | 1.06 | | -0.08 – 2.21 | 0.069 | -0.27 | | -1.16 – 0.61 | | 0.536 |
| Reasoning Speed | -0.09 | | -1.41 – 1.22 | 0.889 | 0.00 | | -0.87 – 0.88 | | 0.992 |

## *Note: AUT Fluency = Number of responses produced.*
